# Identifying Common Patterns in the Time of Day of Mindfulness Meditation Associated with Long-Term Maintenance

**DOI:** 10.3390/bs15030381

**Published:** 2025-03-18

**Authors:** Rylan Fowers, Aurel Coza, Yunro Chung, Hassan Ghasemzadeh, Sara Cloonan, Jennifer Huberty, Vincent Berardi, Chad Stecher

**Affiliations:** 1 Select Health, Murray, UT 84123, USA; rylanf@gmail.com; 2 Corporate Engagement & Strategic Partnerships, Arizona State University, Tempe, AZ 85281, USA; aurel.coza@asu.edu; 3 College of Health Solutions, Arizona State University, Phoenix, AZ 85004, USA; yunro.chung@asu.edu (Y.C.); hassan.ghasemzadeh@asu.edu (H.G.); 4 Department of Psychology, University of Georgia, Athens, GA 30602, USA; sara.cloonan@uga.edu; 5 Fit Minded Inc., Phoenix, AZ 85032, USA; jen@jenhubertyphd.com; 6 Crean College of Health and Behavioral Science, Chapman University, Orange, CA 92866, USA; berardi@chapman.edu

**Keywords:** behavior maintenance, mindfulness meditation, temporal consistency, habit formation, app engagement

## Abstract

Forming a habit of practicing mindfulness meditation around the same time of day is one strategy that may support long-term maintenance and in turn improve physical and mental health. The purpose of this study was to identify common patterns in the time of day of meditation associated with long-term meditation app use to assess the importance of temporal consistency for maintaining meditation over time. App usage data were collected from a random sample of 15,000 users who had paid for an annual membership to a commercial meditation app in 2017. We constructed three measures of temporal consistency in the time of day of meditation sessions in order to categorize users into one of three behavioral phenotypes: Consistent, Inconsistent, or Indeterminate. Panel data models were used to compare temporal consistency across the three phenotypes. Of the 4205 users (28.0%) in the final analytic sample, 1659 (39.5%) users were Consistent, 2326 (55.3%) were Inconsistent, and 220 users (5.23%) were Indeterminate. Panel models confirmed that temporal consistency had contrasting relationships with meditation maintenance among these three phenotypes (*p* < 0.01). These findings revealed that temporal consistency was associated with meditation maintenance for less than half of app users, which suggests that other behavioral mechanisms in addition to temporally consistent habits can support meditation app use over time. This has important implications for researchers and policymakers trying to promote the maintenance of meditation and other complex health behaviors, such as increased physical activity and healthier diets.

## 1. Introduction

Mindfulness meditation has been linked to numerous mental and physical health benefits such as reduced stress, improved anxiety and depressive symptoms, lower blood pressure, chronic pain management, and greater overall well-being (Bostock et al., 2019; Herman et al., 2017; Pokorski & Suchorzynska, 2018; Ponte Márquez et al., 2019; Saeed et al., 2019). Past research has demonstrated the importance of maintaining a consistent meditation practice over time in order to maximize the health benefits associated with mindfulness meditation (Shen et al., 2020; Tang et al., 2012). Unfortunately, like many other health behaviors, long-term maintenance of meditation is a significant challenge for many individuals (Forbes et al., 2018; Howells et al., 2016; Torous et al., 2020). The rise of mobile health platforms over the last decade has increased the accessibility of meditation practice while also offering comparable health benefits to traditional face-to-face meditation interventions (Gál et al., 2021; Longyear & Kushlev, 2021; Muñoz et al., 2016). Despite this, research has shown that less than 10% of health app users maintain their engagement with an app over time (Baumel et al., 2019). High dropout rates are also common in meditation intervention settings, with the average attrition rate estimated to be between 26% and 43% (Meyerowitz-Katz et al., 2020; Torous et al., 2020). These findings underscore the importance of identifying effective behavioral strategies to help individuals maintain an app-based meditation practice over time and attain the associated health benefits of consistent meditation practice. Given the large and growing mental health crisis and the associated economic costs (Greenberg et al., 2021; Stecher et al., 2024), maintaining app-based meditation is a promising solution to address this important public health issue.

Habits are one potential strategy for maintaining app-based meditation practice over time. The habit formation process occurs by repeatedly performing a target behavior in response to the same contextual cue until the behavior becomes automatically, or reflexively, initiated upon encountering the contextual cue (Gardner, 2015; Lally et al., 2010; Verplanken & Melkevik, 2008). Habits have been shown to support many health behaviors by reducing the cognitive effort required to perform the behavior (Lally et al., 2011; Wood & Neal, 2016), allowing the behavior to persist despite waning motivation or distractions (Galla & Duckworth, 2015; Gardner et al., 2011; Rebar et al., 2014). Habit-based interventions have been used to promote the maintenance of several health behaviors including teeth brushing and flossing (Judah et al., 2013; Wind et al., 2005) and medication adherence (Brooks et al., 2014; O’Carroll et al., 2013; Phillips et al., 2016). This line of research has also shown that consistently performing a behavior around the same time of day plays a key role in the habit-formation process (Berardi et al., 2023; Schumacher et al., 2019; Stecher et al., 2021; van der Weiden et al., 2020) since people often encounter the same contextual cues around similar times in the day (Gardner et al., 2012). Thus, a pattern of consistently timed mindfulness meditation may be indicative of a habit and an important strategy for long-term mindfulness meditation maintenance.

The purpose of this research was to investigate the role of temporally consistent habits for maintaining app-based meditation among a large sample of individuals who subscribed to Calm, a commercially available mindfulness meditation app. While existing research has demonstrated the important role of temporally consistent habits for maintaining simple health behaviors, such as medication adherence (Phillips et al., 2013, 2023), this study provides a unique investigation into the association of temporal consistency and maintained app-based meditation. The Calm app provides an ideal setting for this research, as prior studies have described the significant health benefits of maintaining the use of the Calm app (Huberty et al., 2021, 2019a, 2019b), but to date, research has not uncovered the successful strategies used by real-world users who have maintained their app-based meditation practice. Using objective app usage data from over 15,000 subscribers with varying levels of maintenance, we implemented a novel technique to identify patterns in the time of day of meditation app use that were associated with long-term meditation maintenance. By examining the role of temporally consistent habits for maintaining meditation app use, researchers, public health policymakers, and app developers will be able to develop more effective approaches for supporting the maintenance of meditation, which in turn may offer solutions for maintaining other complex health behaviors, such as increased physical activity and healthier diets.

## 2. Materials and Methods

### 2.1. Participants and Procedure

Longitudinal behavioral data were collected from the mindfulness meditation app Calm, which had over 4 million paying subscribers at the time of data collection. The data used in this analysis came from a sample of 15,000 randomly selected new users who paid for their first annual membership in 2017. The sample was also selected so that roughly one-quarter of users never renewed their annual subscription, one-quarter of users renewed once, one-quarter renewed twice, and one-quarter renewed three times, which means the data potentially span from 2017 to 2021 for some users. The sample was selected in this way to ensure that we would observe different durations of app use, and thus we would be able to describe the time-of-day patterns associated with long-term maintenance. The data contain the start time and duration of all app sessions performed by each user, but no demographic information was collected by the app. Because this was a retrospective observational study, informed consent was not directly obtained from participants. However, when users subscribe to the Calm app, they provide consent for their data to be used for research purposes. This study was approved by the Arizona State University Institutional Review Board (Study #: 00012530).

### 2.2. Measures

Each participant’s data were split into four-week chunks beginning on their membership start date. A dichotomous minute-level time series was created for each chunk, with a 1 indicating a minute where a meditation session was completed and a 0 otherwise. We then used these minute-level time series to calculate three behavioral measures of temporal consistency: (1) dynamic time-warping (DTW) distance, (2) the variance of mindfulness meditation session start times, and (3) entropy.

#### 2.2.1. Dynamic Time-Warping Distance

Temporal consistency between the first 14 days and the last 14 days of the same 4-week chunk was computed using a DTW algorithm, which calculates an adjusted distance measure that allows for flexibility in the timing of similar data patterns. That is, the DTW distance between two time series does not increase if there are similar patterns of behavior that occur at slightly different time points. For example, meditation for 10 min at 9:00 am would be treated equivalently to meditation for 10 min at 9:15 am on another morning. This flexibility is important for capturing the performance of a habitual behavior that is triggered by a cue with some variability in time of day, such as coffee or breakfast in the morning. DTW was calculated using the Python (version 3.12.2; Python Software Foundation; Fredericksburg, VA 22401 USA) software package ‘dtw’ (Giorgino, 2009), where we used the ‘Sakoechiba’ window type with a window size set to 2 (to allow for 2 h of flexibility) and the step pattern of ‘symmetric1.’ We adjusted the DTW distance measure by penalizing consecutive time intervals with 0 meditations to differentiate between temporally consistent app use and consistent non-app use. The adjusted DTW was defined as 1 when there were no sessions on consecutive 14-day periods, and all other DTW distances were scaled by dividing by the total number of 2 h windows that contained any minutes of meditating with the app on the previous day (plus 1 to avoid division by 0). This scaling was used so that the penalized distance of 1 would be high relative to the DTW distance calculated on days with actual meditation app use. The adjustment on DTW was conducted as follows:

(1) $$Adjusted\;DTW=\left\{\begin{array}{cc} 1, & \mathrm{If}\;\mathrm{no}\;\mathrm{activity}\;\mathrm{in}\;\mathrm{consecutive}\;14\;\mathrm{day}\;\mathrm{periods}\\ \frac{DTW}{2hrs+1}, & \mathrm{Otherwise} \end{array}\right.$$

where *2hrs* is the number of 2 h windows with any minutes of meditation using the app on the previous day.

#### 2.2.2. Session Timing Variance

Our second behavioral measure of temporal consistency was the variance of meditation session start times. We calculated within each 4-week chunk by first transforming session start times into circular distances to avoid overestimating distances between days (e.g., 11 PM on one day and 2 AM the next day is correctly calculated as a 3 h difference). The resulting variance in session start times was calculated as follows:

(2) $$\sigma_{Time}^{2}=\frac{\sum {\left(d(t_{i,}\bar{t})\right)}^{2}}{n-1}$$

where $t_{i}$ represents the start time of a meditation session i, $\bar{t}$ is the average start time, and $d(t_{i,}\bar{t})$ is the Cartesian distance on the 24 h clock, i.e., $d\left(t_{i,}\bar{t}\right)=\min\left(\left|\frac{2\pi t_{i}}{24}-\frac{2\pi \bar{t}}{24}\right|,\;2\pi -\left|\frac{2\pi t_{i}}{24}-\frac{2\pi \bar{t}}{24}\right|\right).$

#### 2.2.3. Entropy

The entropy of meditation sessions was calculated for each 4-week chunk, which captures the uncertainty in mindfulness meditation timing as follows:

(3) $$H=-\sum_{i=1}^{4}P(x_{i})\log P(x_{i}),$$

where H is the entropy, and $P(x_{i})$ is the empirically calculated probability of meditating during time window i, where $i=\left\{\mathrm{morning},\;\mathrm{midday},\;\mathrm{evening},\;\mathrm{latenight}\right\}$. H can take values between 0 and log 0.25 (=1.38), with 0 representing meditating exclusively in a single time window (i.e., temporal consistent session timing) and log 0.25 representing an equal probability of meditating during each of the four windows (Berardi et al., 2023). Each block of time was defined as being in the morning (between 4:00 A.M. and 10:00 A.M.), midday (between 10:00 A.M. and 4:00 P.M.), evening (between 4:00 P.M. and 10:00 P.M.), or late night (between 10:00 P.M. and 4:00 A.M.).

### 2.3. Statistical Analysis

We used Least Absolute Shrinkage and Selection Operator (LASSO) regression models to predict future app use (i.e., the number of meditation sessions performed in each 4-week chunk) based on our three temporal consistency measures calculated over prior 4-week chunks. That is, the dependent variable of our models was the number of meditation sessions during a 4-week chunk and the independent variables were the three temporal consistency measures (DTW, variance, and entropy) calculated over prior 4-week chunks. To determine the optimal number of prior chunks to use for calculating our independent variables, we estimated LASSO models with systematically varying amounts of past data and calculated the adjusted *R*^2^ as a measure of goodness-of-fit. As we added more past chunks of data to the model, the number of complete observations decreased, so we selected the model with the highest adjusted *R*^2^ to optimally balance the tradeoff between more independent variables and a smaller set of complete observations. Based on the adjusted *R*^2^, it was determined that the model with temporal consistency calculated over the previous two chunks of data yielded the best goodness-of-fit.

In order to determine each user’s behavioral phenotype, we followed three main steps. First, we used our temporal consistency measures calculated over the previous two chunks of data to predict future app use for each user. Second, the coefficients from these user-level models were used to categorize our sample into three phenotypes. Finally, we estimated models within each of the three phenotypes to confirm our categorization captured the intended behavioral patterns. For a more generalizable discussion of this approach that can be readily applied to other types of longitudinal health behavior data, please see the Supplemental Materials.

To complete these three steps, we first used 10-fold cross-validation to determine a global regularization penalty for the LASSO models of future app use. Then, we fit separate LASSO models of future app use for each user. The purpose of the LASSO models was to classify users based on the relative predictive ability of each temporal consistency metric, which was determined based on the size of the sum of all statistically significant coefficients for a given measure. All measures were standardized to have the same scale, and the temporal consistency measure with the largest estimated association to future app use was used to categorize each user into one of three behavioral phenotypes: (a) Consistent, (b) Inconsistent, or (c) Indeterminate. Users whose largest association with future app use was negative (i.e., more temporal consistency was correlated with greater future meditation) were labeled as Consistent, while those with a positive largest association (i.e., less temporal consistency was correlated with greater future meditation) were labeled as Inconsistent. Users whose model fit was poor (i.e., in the bottom 10th quantile of adjusted *R*^2^ values) were labeled as Indeterminate.

Finally, we assessed the quality of our behavioral pattern detection process by fitting separate panel regression models predicting future app use among the three behavioral phenotypes using our three temporal consistency measures. All independent variables were standardized. The models also estimated fixed effects for each user and included controls for the number of days with any app use and the portion of app use that occurred during COVID-19 lockdowns to improve model fit. The sign and significance of the estimated associations between the temporal consistency measures and future app use among each phenotype were assessed to verify our categorization process (e.g., the Consistent group was expected to show a positive association between increased consistency and future app use). Statistical significance was set at the α = 0.05 level.

## 3. Results

**Figure 1:**
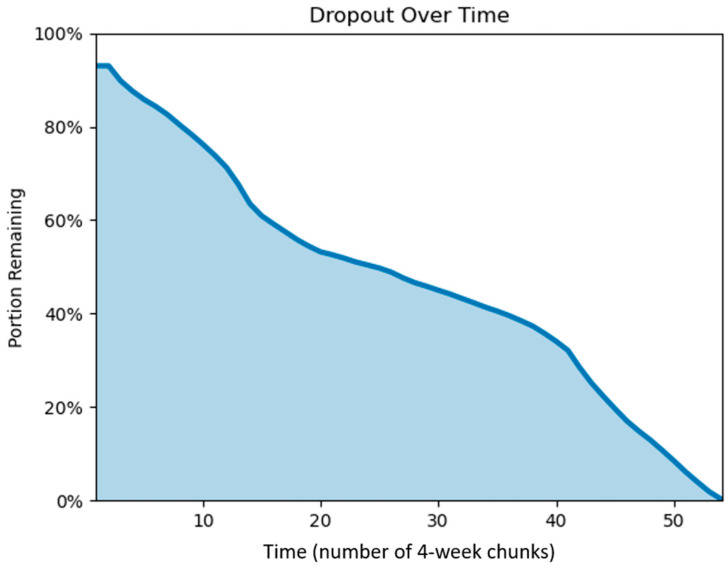
Mindfulness meditation app abandonment (or dropout) after subscribing. The percent of users with any app use in a future chunk of data. Chunk refers to the 4-week interval from the start of users’ app subscriptions.

The dataset began with 15,000 randomly selected users who had initiated their first annual membership with Calm in 2017. Three-quarters of the sample was selected because they renewed their subscription at least once, which was used as a proxy for maintained app use. However, many users were not using the app and had their subscriptions automatically renewed. This can be observed in the high dropout rates following the start of users’ initial subscriptions. As seen in Figure 1, there was a steep decline in the percentage of users who used the app in any future chunk of data. By chunk 15 (i.e., week 60), only 60.9% of users used the app in at least one future chunk of data. By chunk 30 (i.e., week 120), this percentage dropped to 45.0%, and by chunk 45 (i.e., week 180), this number dropped to less than 20.0%.

**Figure 2:**
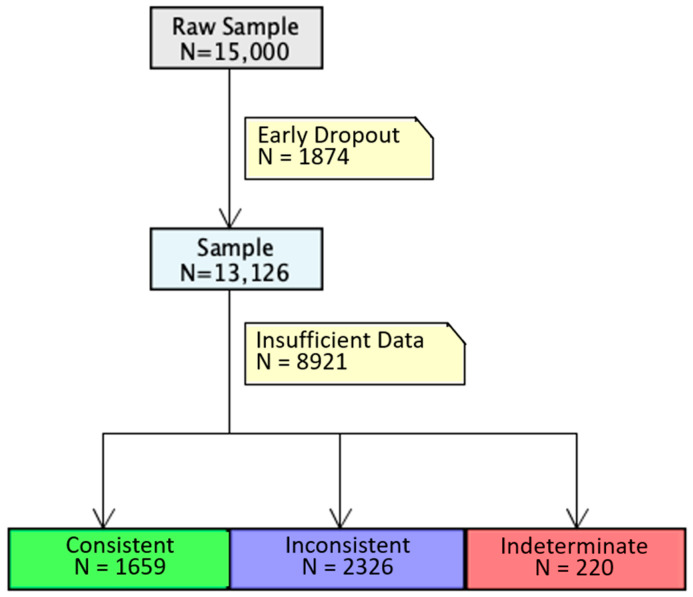
Flow chart of sample size. After determining the minimum data requirements, 1874 individuals were dropped due to insufficient data. When fitting models for each individual, an additional 8921 were dropped due to sporadic app use that prevented the calculation of our temporal consistency measures and/or the estimation of our model of future app use.

A significantly smaller sample of users was categorized into our three behavioral phenotypes than was contained in the original dataset since many users had insufficient data. Figure 2 describes how the sample size was reduced throughout this pattern detection process. First, 1874 users were dropped because they had less than three full chunks worth of data before they dropped out of the sample. Then, 8921 users were dropped because they had infrequent usage data, which prevented the estimation of our model of future app use. This was largely driven by our inaccurate method of sample selection, which relied on subscription renewal as a proxy for maintaining some degree of meaningful app use. Instead, many users displayed highly sporadic app use that made calculating temporal consistency infeasible and/or estimating our models of future app use impossible.

Based on the relative size of the significant coefficients in each user’s model of future app use, 1659 users were categorized in the Consistent phenotype, which indicated that the more consistent their mindfulness meditation, the more sessions they were likely to perform in the future. Additionally, this categorization process led to 2326 users being categorized as Inconsistent and the final 222 users as Indeterminate. Those who were categorized as Indeterminate had poor model fits when predicting their future app use; i.e., the R^2^ values were in the bottom 10.0% quantile (R^2^ < 0.56). Due to the poor model fit, it was unclear if consistency or inconsistency was more strongly associated with these users’ future app use.

Descriptive statistics for our three temporal consistency measures across the three behavioral phenotypes can be found in Table 1. The values indicate the average coefficient for that variable for the regression models calculated for each individual user in our sample. The average coefficients for our temporal consistency measures among the Consistent group were all negative, indicating that more temporal consistency among these users was associated with more future app use. Average coefficients for the Inconsistent group were all positive, indicating that less consistency among these users was associated with more future app use. For the Consistent group, roughly 45.1% of individuals (*n* = 748/1659) had their biggest estimated association with future app use from the adjusted DTW measure (*M* = −6.85, *SD* = 19.53). For those in the Inconsistent group, roughly 46.3% of individuals (n = 1076/2326) had their biggest estimated association with future app use from entropy (*M* = 7.57, *SD* = 10.33).

Table 2 presents the Pearson correlation coefficients estimated between each pair of our three temporal consistency measures. All correlation coefficients were positive, which confirms that they measured similar attributes of behavior. However, the adjusted DTW was only weakly correlated with entropy, and the variance in time was moderately correlated with adjusted DTW and entropy (Papageorgiou, 2022). The relatively low strength of these correlations indicates that these three measures describe unique features of temporal consistency and help to justify the inclusion of all three measures in our panel regression models of future app use.

Table 3 presents the results from the panel regression models in which the number of future meditation sessions was regressed onto our three temporal consistency measures among the three phenotypes. We also included lagged versions of our temporal consistency measures in these models. For the Consistent group, the number of future sessions was negatively associated with all three temporal consistency measures, with entropy having the strongest negative association with future app use, *b* = −0.49, *SE* = 0.08, *p* < 0.001. For the Inconsistent group, the number of future sessions was positively associated with all three temporal consistency measures, with session timing variance having the largest positive association with future app use, *b* = 0.49, *SE* = 0.06, *p* < 0.001. None of the temporal consistency measures were significantly associated with the number of future sessions for those categorized as Indeterminate.

Descriptive statistics for meditation app use across the three behavioral phenotypes can be found in Table 4. Users in the Consistent group meditated with the app for an average of 34.62 (*SD* = 11.79) consecutive chunks (approximately 138 weeks), completed an average of 8.05 (*SD* = 13.55) meditation sessions in each chunk, and meditated with the app on an average of 5.57 (*SD* = 8.35) days per chunk. Users in the Inconsistent group meditated with the app for an average of 33.41 (*SD* = 12.94) consecutive chunks, completed an average of 8.15 (*SD* = 13.62) meditation sessions per chunk, and meditated with the app on an average of 5.67 (*SD* = 8.45) days per chunk. While the Indeterminate phenotype maintained their mindfulness meditation the longest (*M* = 42.57, *SD* = 5.72), users in this phenotype performed fewer meditation sessions per chunk (5.69, *SD* = 10.95; *p* < 0.001) and fewer days with any meditation sessions per chunk (4.02, *SD* = 6.99; *p* < 0.001). The *p*-values for *t*-tests comparing these descriptive statistics between the consistent and inconsistent phenotypes are presented in column 4 of Table 4. Based on these comparisons, the Consistent phenotype maintained their mindfulness meditation for roughly 1.2 chunks longer (approximately 5 weeks) than the Inconsistent group (*p* = 0.003). Otherwise, both the Consistent and Inconsistent phenotypes had a similar average number of meditation sessions per chunk (*p* = 0.184), and a similar variance in the number of meditation sessions per chunk (*p* = 0.496). While the difference in the average number of days with any meditation per chunk was significant (*p* = 0.034), the magnitude of the difference was less than 0.1 days.

**Figure 3:**
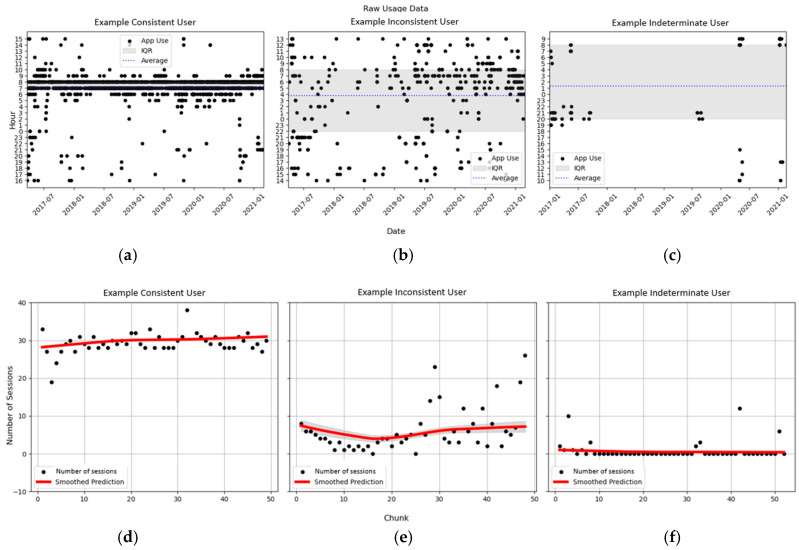
Typical meditation app use patterns for each behavioral phenotype. Top plots show the start time of each app session for a: (**a**) Consistent user, (**b**) Inconsistent user, and (**c**) Indeterminate user. Bottom plots show the total number of app session by chunk for a: (**d**) Consistent user, (**e**) Inconsistent user, and (**f**) Indeterminate user. Chunk refers to the 4-week interval from the start of users’ app subscriptions.

Figure 3 illustrates typical usage patterns from the Consistent, Inconsistent, and Indeterminate phenotypes. In the top half of Figure 3, the time of day of each meditation session is plotted by day, and the bottom of Figure 3 shows the number of meditation sessions by a chunk of data along with the user’s panel regression model predictions for the number of meditation sessions smoothed over the full time period. This figure shows that the panel regression models did a sufficient job of fitting the number of meditation sessions for each phenotype. Importantly, the figure also demonstrates that important differences existed in users’ patterns in the time of day of mindfulness meditation sessions. The consistent user (Figure 3a) is characterized by performing mindfulness meditation at a similar time of day for their entire period of app use. Specifically, this user favored performing mindfulness meditation at or around 7 AM every day. Meanwhile, the inconsistent user (Figure 3b) is characterized by constant but variable meditation session start times. In other words, while this user did frequently perform mindfulness meditation, there was no clear pattern in the time of day. Lastly, the indeterminate user (Figure 3c) is characterized by sparse use. Although this user maintained minimal app use for at least as long as the other two users, there were several long gaps with no app use. These three examples also help to demonstrate what behavioral patterns result in low (Figure 3d), medium (Figure 3e), and high (Figure 3f) values for our temporal consistency measures. Specifically, the average chunk of data for the user (Figure 3d) corresponds to a session timing variance of 0.17 (SD 0.25), entropy of 0.22 (SD 0.08), and an adjusted DTW with the prior chunk of 0.34 (SD 0.54). The average chunk of data for user (Figure 3e) corresponds to a session timing variance of 1.34 (SD 2.14), entropy of 0.52 (SD 0.38), and an adjusted DTW with the prior chunk of 1.25 (SD 1.85), and the average chunk of data for user (Figure 3f) corresponds to a session timing variance of 4.88 (SD 6.73), entropy of 0.73 (0.64), and an adjusted DTW with the prior chunk of 2.29 (SD 3.19).

In order to better understand the behavioral patterns that are associated with future app use, Table 5 compares the proportion of meditation app use by time of day between the Consistent and Inconsistent phenotypes. For both phenotypes, the most popular time window for mindfulness meditation was late at night (10 PM–4 AM), with each phenotype performing more than 41.3% of their meditation sessions during that time window. The Consistent phenotype had a slightly higher portion of their meditation sessions performed in the morning compared to the Inconsistent phenotype, but overall, the distribution of meditation timing was very similar between these two behavioral phenotypes.

## 4. Discussion

The purpose of this study was to investigate the role of temporally consistent habits for maintaining app-based mindfulness meditation by identifying common patterns in the time of day of meditation app use that were associated with long-term maintenance. Using objective app usage data collected from a large sample of meditation app subscribers, we implemented a novel technique to (1) classify users into three different behavioral phenotypes based on the consistency in the timing of their meditation sessions (i.e., a) Consistent, b) Inconsistent, or c) Indeterminate) and (2) examine the relationship between these behavioral phenotypes and maintained app use. Over half of the users in our final analytical sample (55.3%; *n* = 2326/4207) were categorized as Inconsistent, 39.5% of users (n = 1659/4207) were categorized as Consistent, and only 4.8% of users (*n* = 202/4207) were categorized as Indeterminate. Moreover, while the Consistent phenotype used the app for a significantly longer amount of time compared to the Inconsistent phenotype (34.6 consecutive chunks vs. 33.4 consecutive chunks), those categorized as Inconsistent reported significantly more days with meditation during each 4-week chunk compared to those categorized as Consistent (8.0 days vs. 8.2 days). These findings suggest that although consistently timed app-based meditation habits may help support maintained app use for some users, many users maintained regular app use over time through other behavioral mechanisms.

There are several behavioral theories and empirical research findings that suggest temporally consistent habits could have a large role in maintaining app-based meditation practices. The psychology literature defines habits as non-deliberative behaviors that are performed with little to no cognitive effort or self-regulatory resources (Verplanken, 2006), making habits an important mechanism for maintaining behaviors over time. The Multi-Process Action Control model also identifies habits as one of the primary mechanisms for behavioral maintenance (Rhodes, 2021). Additionally, empirical research has often found that habit strength predicts behavioral maintenance more than conscious, deliberative processes, such as intentions, beliefs, or attitudes (Phillips et al., 2016; Thorneloe et al., 2018). Empirical research has also found that habits are frequently performed in the same location around the same time of day (Gardner et al., 2012). For example, temporal consistency is a strong indicator of medication adherence habits (Phillips et al., 2020, 2023), and temporally consistent habits are a key strategy used by successful long-term medication adherers (Brooks et al., 2014). However, few app-based interventions or observational studies have examined the role of temporally consistent habits for maintaining app engagement, particularly meditation app use. Thus, in this study, we chose to investigate the potential role of temporally consistent habits for maintaining meditation app usage.

In order to study temporally consistent meditation habits, we calculated and standardized three measures of temporal consistency: dynamic time-warping (DTW) distance, session timing variance, and entropy. For all three standardized metrics, larger positive scores indicated less consistent timing of meditation app use, while smaller negative scores indicated more consistent timing of app use. For those categorized into the Consistent group, our three measures of temporal consistency were negatively associated with future meditation app use, with adjusted DTW emerging as the strongest predictor of future app use. By definition, this negative association implied that using the app around the same time of day (i.e., lower adjusted DTW) was associated with more app-based meditation practice over time. Users categorized into the Consistent group also maintained their mindfulness meditation practice significantly longer than those categorized as Inconsistent, although the difference in duration was only approximately 5 weeks (i.e., 1.2 chunks). Future research should aim to better characterize these users and collect more information about the location and contextual cues for these temporally consistent habits, which would help to further inform the targeting and design of habit-based mindfulness meditation interventions.

While temporally consistent habits may have supported maintained app use for 39.5% of our sample, the majority (55.3%) of the sample was categorized into the Inconsistent group where our temporal consistency measures were positively associated with future app use, with session timing variance emerging as the strongest predictor of future app use. That is, for individuals in the Inconsistent group, more variance in the time of day they used the app (i.e., greater session timing variance) was associated with more app-based meditation over time. While the Consistent group maintained their app use significantly longer than the inconsistent group, those in the Inconsistent group recorded significantly more days with any app use during each chunk. These differences in the amount of meditation between the Consistent and Inconsistent groups were qualitatively small though and suggest that both temporal consistency and temporal inconsistency characterized successful strategies for maintaining meditation app use in our sample.

One explanation for our finding that temporal inconsistency was displayed by the majority of users who maintained meditation app use is that they could have formed a habit that was cued at variable times of day. For example, it could be that users in the Inconsistent group formed a habit of meditating when they felt stressed, and the feeling of stress could have sporadically occurred throughout the day. Similarly, meditation habits could have been cued by other mental health symptoms, such as anxiety or depressive thoughts, which would explain how temporal inconsistency was associated with maintained meditation app use.

Another reason for the association between temporal inconsistency and maintained meditation app use for those in the Inconsistent group could be that other behavioral mechanisms, besides habits, were supporting meditation practices. A wide range of mechanisms for behavioral maintenance have been proposed across the Health Belief Model (Becker & Maiman, 1975), the Transtheoretical Model (Prochaska & Velicer, 1997), and the Social Cognitive Theory (Bandura, 1986). Among these mechanisms are intrinsic motivation, self-efficacy, and affective processes. Intrinsic motivation, defined as an individual’s motivation to engage in behavior due to personal enjoyment and interest, has been associated with initiating and maintaining several different health behaviors (Ntoumanis et al., 2021), including meditation (Cardeña et al., 2015; Ryan et al., 2021). Self-efficacy, which refers to an individual’s perceived competence or confidence in performing goal-directed behaviors (Bandura, 1977), has also been cited as a key determinant of health behavior initiation and maintenance (Amireault et al., 2013; Schwarzer, 2008). Affective processes have also been shown to play an important role in the maintenance of various health behaviors, including meditation (Cohn & Fredrickson, 2010; Dunton & Vaughan, 2008; Van Cappellen et al., 2018). For example, one study found that novice meditators who experienced higher levels of positive affect during meditation were over four times more likely to maintain their medication practice 15 months later (Cohn & Fredrickson, 2010). Other mechanisms of behavioral maintenance may be also present such as identity, learning, emotion regulation, and attention and cognitive control (Schuman-Olivier et al., 2020). These other mechanisms may be particularly important for complex behaviors since empirical research has shown that habits play a weaker role in maintaining complex behaviors such as physical activity (Phillips et al., 2024).

Taken together, our findings suggest that consistently timed meditation habits may not be successful in maintaining meditation app use for the majority of individuals. Some research has theorized that the efficacy of the habit-formation process is largely dependent on the complexity of the behavior (Phillips & Mullan, 2023), which would help explain the success of temporally consistent habit-based interventions for relatively simple behaviors such as medication adherence (Brooks et al., 2014; O’Carroll et al., 2013; Phillips et al., 2016) and teeth-brushing (Judah et al., 2013; Wind et al., 2005). For more complex behaviors like meditation, there may be other mechanisms underlying long-term maintenance, such as intrinsic motivation, identity, and/or affective processes. More research is needed to better understand the relative role of temporally consistent habits versus these other behavioral mechanisms for maintaining mindfulness meditation (Bryan et al., 2021; Miller, 2019). These findings will ultimately enable researchers and policymakers to design interventions that can enable individuals to successfully attain the health benefits of mindfulness meditation. In addition to meditation, this research agenda can help uncover behavioral maintenance mechanisms for other complex health behaviors, such as physical activity and diet, which also generate important public health benefits.

### Limitations

This study utilized high-frequency longitudinal behavioral data from a popular commercial mobile health app, which increased the real-world applicability and generalizability of the results. However, there are several limitations to these data that should be addressed. First, since the data come from a single mindfulness meditation app that contained a range of meditation content and other app-based behavioral supports, such as reminders and activity tracking features, the time-of-day patterns observed in this study may not apply to other mindfulness meditation apps. Additionally, the users of this mindfulness meditation app may not be comparable to the users of other health apps. Second, the data did not contain information about the location, context, or preceding behaviors for each mindfulness meditation session, which is necessary to accurately identify habits. Specifically, the Inconsistent phenotype in our sample could have formed a habit based on an inconsistently timed contextual cue, such as experiencing stress, which our data would not be able to capture. Third, user characteristics and affective states were not available, which would provide important information for better targeting future interventions and for understanding the behavioral mechanisms underlying mindfulness meditation maintenance. Finally, several measurement and data transformation decisions were made that could have influenced the results, such as the selection of our temporal consistency measures and dividing the time series into 4-week chunks of data. Additional research is needed to fully understand how these analytical choices influence the results of our pattern detection process.

## 5. Conclusions

This study used longitudinal behavioral data to identify common patterns in the time of day of mindfulness meditation that were associated with long-term maintenance. Over half of users maintained inconsistent patterns in the timing of their meditation app use, which is contrary to findings for other health behaviors, such as medication adherence, where maintaining temporal consistency is more common (Brooks et al., 2014; Phillips et al., 2023). This finding suggests that temporally consistent habits are only one possible mechanism for maintaining app-based meditation, and future research should examine the role of other mechanisms for designing interventions to help people attain the health benefits of long-term meditation. Importantly, identifying successful strategies for maintaining app-based meditation is a promising public health solution for addressing the significant and growing mental health crisis and may offer strategies for maintaining other complex health behaviors, such as increased physical activity and healthier diets.

## Figures and Tables

**Table 1:** Descriptive statistics of the model coefficients by behavioral phenotype ^1^.

Variable	Consistent		Inconsistent		Indeterminate	
	*M* (*SD*)	*n* (%)	*M* (*SD*)	*n* (%)	*M* (*SD*)	*n* (%)
Adjusted DTW	−6.845	748	7.16	314	−0.82	87
	(19.53)	(45.1%)	(12.29)	(13.5%)	(3.44)	(39.6%)
Entropy	−13.39	415	7.57	1076	1.18	73
	(63.80)	(25.0%)	(10.34)	(46.3%)	(6.69)	(33.2%)
Variance of time	−38.23	496	13.81	936	−0.01	60
	(253.50)	(29.9%)	(38.33)	(40.4%)	(0.70)	(27.3%)
Total	1659		2326		220	

^1^ Descriptive statistics of the effect of each temporal consistency measure by behavioral phenotype. Individuals were labeled as Consistent if the strongest associations with future app use were negative, Inconsistent if the strongest associations were positive, and Indeterminate if the model fit was poor (below the 10th quantile). Each cell displays the mean (and standard deviation in parentheses) of the estimated coefficients for the temporal consistency measure indicated in the row labels on our independent variable (future app use).

**Table 2:** Pearson correlation between the three temporal consistency measures ^1^.

Measure	Adjusted DTW	Entropy	Variance of Time
Adjusted DTW	1		
Entropy	0.34	1	
Variance of time	0.43	0.52	1

^1^ This table displays the Pearson correlation coefficient between the three temporal consistency measures (adjusted DTW, entropy, and variance of time) estimated over all available chunks of data for our analytical sample (*n* = 4205).

**Table 3:** Association between temporal consistency measures and the number of future app sessions by behavioral phenotype ^1^.

Variable	Consistent (*n* = 1659)	Inconsistent (*n* = 2326)	Indeterminate (*n* = 220)
	*b* (*SE*)	*b* (*SE*)	*b* (*SE*)
Adjusted DTW	−0.25 ***	0.37 ***	−0.02
	(0.06)	(0.05)	(0.09)
Lagged adjusted DTW	−0.13 *	0.21 ***	−0.03
	(0.06)	(0.04)	(0.09)
Entropy	−0.49 ***	0.35 ***	0.18
	(0.08)	(0.08)	(0.16)
Lagged entropy	−0.29 ***	−0.08	0.33
	(0.07)	(0.07)	(0.18)
Variance of time	0.11	0.49 ***	0.01
	(0.06)	(0.06)	(0.10)
Lagged variance of time	0.04	0.28 ***	−0.06
	(0.05)	(0.05)	(0.10)

^1^ This table displays the regression coefficients (with standard errors in parentheses) for panel regression models of future app use, measured by the number of mindfulness meditation sessions, estimated within each of the three behavioral phenotypes indicated in the column headings. All models included the same covariates and used fixed effects. Standard errors are listed in parentheses. * *p* < 0.05; *** *p* < 0.001.

**Table 4:** Descriptive statistics of meditation app use by behavioral phenotype ^1^.

Variable	Consistent	Inconsistent	Indeterminate	Consistent vs. Inconsistent	Difference Across Groups
	*M* (*SD*)	*M* (*SD*)	*M* (*SD*)	*p*-Value	*p*-Value
Consecutive Chunks with Any Use	34.62	33.41	42.57	0.003	<0.001
	(11.79)	(12.94)	(5.72)		
Days with Any Use per Chunk	5.57	5.67	4.02	0.034	<0.001
	(8.356)	(8.45)	(6.99)		
Portion of Use During COVID (2020)	0.19	0.19	0.24	0.362	<0.001
	(0.39)	(0.38)	(0.42)		
Number of Meditation Sessions per Chunk	8.05	8.15	5.69	0.184	<0.001
	(13.55)	(13.62)	(10.95)		
Variance in Number of Meditation Sessions per Chunk	0.02	0.02	0.01	0.496	<0.001
	(0.04)	(0.04)	(0.04)		
Individual Model *R*^2^	0.84	0.85	0.47	0.021	<0.001
	(0.14)	(0.13)	(0.06)		
Number of Users	1659	2326	220		
Observations	58,579	79,389	9555		

^1^ Descriptive statistics of mindfulness meditation behavior by phenotype. Statistical comparisons of the indicated variables between phenotypes were performed using *t*-tests.

**Table 5:** Time of day of mindfulness meditation by behavioral phenotype ^1^.

Time of Day	Consistent	Inconsistent
Morning	29.8%	26.6%
Midday	11.9%	13.7%
Evening	16.3%	18.4%
Late Night	41.9%	41.3%

^1^ The percent of mindfulness meditation sessions by time-of-day windows (morning, midday, evening, and late night) for the Consistent and Inconsistent phenotypes. Morning is defined as 4:00 A.M. to 10:00 A.M.; midday as 10:00 A.M. to 4:00 P.M.; evening as 4:00 P.M. to 10:00 P.M.; and late night as 10:00 P.M. to 4:00 A.M.

## Data Availability

The data that support the findings of this study are available from Calm, but restrictions apply to the availability of these data, which were used under license for the current study and so are not publicly available. Data will be made available from the authors upon reasonable request and with permission of the Calm app.

## Supplementary Materials

The following supporting information can be downloaded at: https://www.mdpi.com/article/10.3390/bs15030381/s1, Figure S1: Generalized analytical schematic; Figure S2: Variation in mindfulness meditation app use after subscribing.
