# Supplementary material for: Identifying Common Patterns in the Time of Day of Mindfulness Meditation Associated with Long-Term Maintenance
Source: Behav Sci (Basel). 2025 Mar 18;15(3):381. doi: 10.3390/bs15030381 (PMC11939581; doi:10.3390/bs15030381)
Supplement: Supplementary file 1 [file behavsci-15-00381-s001.zip › behavsci-3300556-supplementary.pdf]

Supplemental Materials

Generalized Pattern Detection Process

We developed a novel process for detecting common patterns in longitudinal behavioral data associated with long-term behavioral maintenance, which is outlined below in Figure S1. To illustrate how this process works, imagine that each person in a high-frequency longitudinal dataset of *N* people has a $M \times T_Y$ matrix of behavioral data, where *M* represents the number of different behaviors that are measured at T_Y sequential time points, where time could be measured in seconds, minutes, or hours. We first split each person's full-time series into shorter sequential time windows of equal length (referred to as chunks of data), e.g., $M \times (T_1 - T_a)$; $M \times (T_{a+1} - T_{2*a})$; $M \times (T_{2*a+1} - T_{3*a})$, where a>0 and 3*a=*Y*. Then, to determine if there was a common time window (or chunk) when behavior significantly changed (e.g., when a large fraction of app users dropped out or shifted to another time of day pattern), we calculated the Euclidian distance between every pair of data chunks (i.e., the square root of the sum of squared differences between corresponding elements of two chunks of data). For each chunk of data, we then found the average distance to all other chunks across all people in the sample and constructed a line plot of the average distance by sequential chunks of data to visually identify changes in behavior over time. If a noticeable change in the distance between chunks occurs, this inflection point represents an important moment when many individuals begin to deviate from their initial behavioral pattern.

It is important to note that the presence of systematic inflections will vary based on the nature of the data as well as the behavior. If an inflection point is present (lines 4-6 in Figure S1a), then descriptive behavioral measures (e.g., number of sessions, total duration of sessions, consistency in time of day of sessions) should be calculated using the chunks of data that occur immediately before and after the inflection point, and regression models can be used to identify the measures that changed the most at this point. If an increase in consistency is found to be the largest relative change at the inflection point, this finding would suggest that

people are forming a mindfulness meditation habit. When no inflection points are present (lines 8-9 in Figure S1a), descriptive behavioral measures should be calculated for all chunks of data, and regression models that use the descriptive behavioral measures to predict behavior during future chunks of data can identify the measures that are most important for understanding behavioral maintenance. Whether observable inflections are present or not, this process will successfully identify the behavioral measures that are important for understanding how behaviors are maintained in a given setting (e.g., is it the overall volume of behavior, frequency of performance, or consistency in the timing of behavior in the day). The remaining parts of the methods section detail how we used this generalized process to detect the behavioral patterns associated with the long-term maintenance of mindfulness meditation.

Algorithm 1: Processing Behavioral Patterns

```
   Result: Categorized Behavioral Patterns
   Input  : Longitudinal Observations
   Output: Pattern Label
1  Step 1: Split data into chunks of time
2  Step 2: Calculate average distance from each chunk to all others
3  Step 3: Visualize distances through time
4  if Systematic Inflection Points then
5  |  Step 4:  Compute descriptive metrics of behavior at the inflection
6  |  Step 5:  Determine metric changes at inflection point
7  else
8  |  Step 4:  Compute descriptive metrics of behavior at each chunk
9  |  Step 5:  Fit regression models for each individual
10 end
```

(**a**)

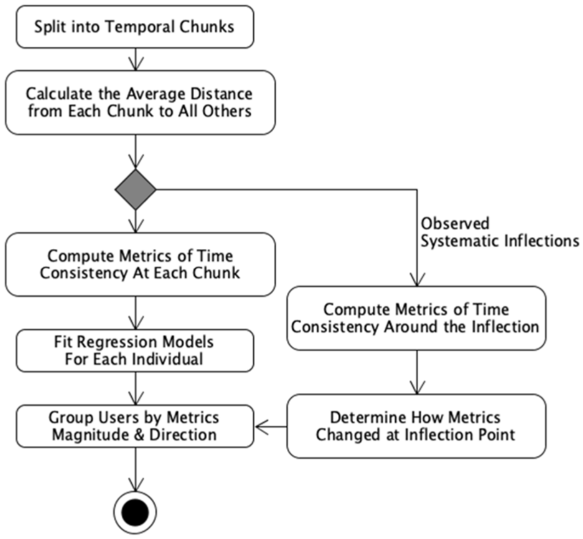

(**b**)

Figure S1. Generalized schematic for characterizing behavioral patterns in longitudinal data: (**a**) (left) the behavioral pattern detection is outlined as a step-by-step algorithm; (**b**) (right) the process flow diagram outlines how to detect inflection points in behavioral data and determine the important behavioral patterns at the inflection points (if applicable).

As seen in Figure S2, there were no clear inflection points in the average distance over all meditation app users in our data.

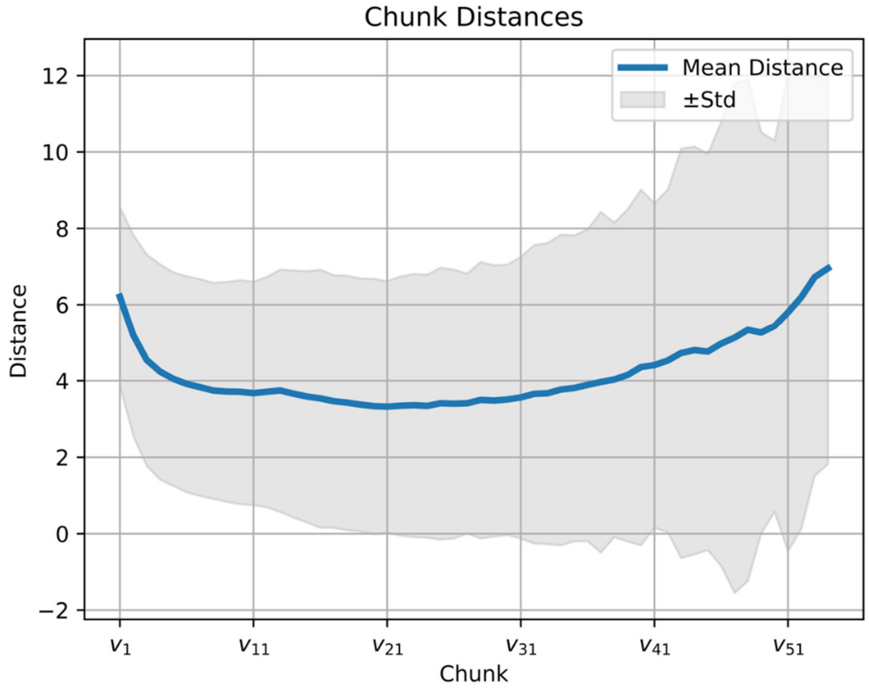

Figure S2. Variation in mindfulness meditation app use after subscribing. The distance between the indicated chunk of data and all other chunks averaged over all users. Chunk refers to the 4-week interval from the start of users' app subscription. V1 indicates the first 4-week chunk, V2 indicates the second 4-week chunk, etc…

Due to the absence of clear inflection points in our data, we used Least Absolute Shrinkage and Selection Operator (LASSO) regression models to predict future app use (i.e., the number of meditation sessions performed in each 4-week chunk) based on our temporal consistency measures calculated over prior 4-week chunks. These temporal consistency measures were calculated for all users and all chunks of data using Steps 4 and 5 in the algorithm shown in Figure S1.
